# Proximal hypospadias: we aren’t always keeping our promises

**DOI:** 10.12688/f1000research.9230.1

**Published:** 2016-09-26

**Authors:** Christopher J. Long, Douglas A. Canning

**Affiliations:** 1 Division of Urology, The Children's Hospital of Philadelphia, Perelman School of Medicine at the University of Pennsylvania, Philadelphia, PA, USA

**Keywords:** hypospadias, hypospadias surgery, proximal hypospadias

## Abstract

Hypospadias surgery is a humbling art form. The evolution of surgical techniques has made distal hypospadias outcomes favorable, but recent publications suggest that our complication rates for proximal hypospadias are much higher than previously reported. To explain these shortcomings, we examine the literature and focus on the lack of standardized documentation, the subsequent inability to objectify the severity of the phenotype, and the underestimation of complications due to lack of long-term follow up. The variability in surgical technique and the fact that the literature abounds with small case series from single institutions also limits our ability to compare outcomes. We believe that the use of standardized and scored phenotype assessments from diagnosis through the extended postoperative period will allow for improved scientific assessment of outcomes. This will facilitate multi-institution collaboration and tabulation of outcomes, allowing rapid data accumulation and assessment for this rare disorder. As surgeons, we must follow boys through puberty into adulthood and must honestly report our results in order to advance our surgical approach to this complicated problem.

## Introduction

Proximal hypospadias, defined by a urethral meatus located at the penoscrotal junction after penile degloving in the operating room, is the most severe manifestation of the hypospadias spectrum. Recent efforts suggest a complication rate that is much higher than previously reported for proximal compared to distal variants ^1– 3^. This high complication rate is worrisome. Many of the complications are not noticed until adulthood, often resulting in considerable compromise and need for late surgery with high complication rates ^4^. The preoperative consent process must include disclosure of these high failure rates. What is more, we must improve our outcomes.

## Background

The hypospadias complex consists of varying degrees of penile curvature (chordee), an incomplete, dorsal hooded foreskin, and a proximal urethral meatus. The ventrum of the penis is often lined with underdeveloped shaft skin, and in many cases the scrotum is displaced anteriorly, creating a penoscrotal transposition, which suggests the potential for disorders of sexual development. In the mildest form of hypospadias, with the urethral meatus in the glans and no chordee, surgical intervention can be avoided with minimal functional consequences. Severe proximal variants, however, result in significant penile curvature that limits sexual and voiding function, which presents a complex surgical entity for the pediatric urologist. If uncorrected, these boys with severe phenotypes suffer from poor body image, a short penis with potentially painful erections, and an inability to direct the urinary stream.

The goals of penile reconstruction of proximal hypospadias are as follows: to allow the boy to void with normal velocity and laminar flow, to obtain satisfactory sexual function with a straight penis, and, from a cosmetic standpoint, to achieve a slit-like meatus with a well-approximated glans. Ultimate surgical success and assessment of these goals cannot be limited to the infant or toddler phase, as sexual function, urethral lumen development, and penile growth are not completed until the late teenage years. Unrepaired or complications after repair of hypospadias can result in a splayed urinary stream that requires one to sit to void and/or painful or awkward sexual function due to penile curvature or shortening. Although no one would consider these concerns life threatening, most would agree that quality of life is compromised for these boys and men, warranting our attention.

Reviewing the history of hypospadias repair evolution reveals tremendous progress. Records from ancient Greece include the first description in which repair consisted of partial penectomy to the level of the ectopic urethral meatus ^5^. Thankfully, technical advancements have since focused upon improved function and cosmesis. The 19 ^th^ century was significant for the addition of important technical elements such as preputial skin flaps, urethroplasty, and multi-layered closure ^5^. The 1980s and 90s were notable for technical advances for distal hypospadias, vastly improving postoperative appearance and function with the introduction of procedures such as meatal advancement and glanuloplasty (MAGPI), glans approximation procedure (GAP), and tubularized incised plate urethroplasty (TIP) ^6– 8^. These “game-changing” procedures drastically improved cosmetic and functional outcomes, markedly elevating surgeon and patient expectations. For a variety of reasons, these expectations have now been extended to proximal repairs, but we often fall short of these expectations.

In 1995, John Duckett tabulated his experiences with the complexity of hypospadias repair to coin the term “hypospadiology” ^9^. Duckett described hypospadias surgery as a humbling process, a time- and energy-consuming task that often confounds the surgeon, highlighting the complex blend of art and science that produces a successful repair. Pediatric urologists with hypospadias experience understand and can relate to these words, and although some progress has been made since this description, much work remains to be done in hypospadiology ^9^.

## Identification of the problem

The surgical advances in the management of distal hypospadias have led to success rates ranging from 85 to 95% ^10– 13^. When one excludes studies including adult patients, small case series, and re-do surgeries, the overall complication rate is <10% ^13^. In contrast, reported rates for proximal hypospadias have been comparatively less favorable, yet acceptable, ranging from 75 to 90% ^14– 16^. Urethrocutaneous fistula, glans dehiscence, and meatal stenosis are the most common complications encountered in hypospadias surgery, each occurring in 5–18% of patients ^17– 19^. Surgical reconstruction for proximal hypospadias is more extensive than for distal variants and, although many approaches exist, they can be broadly characterized by the single- and multi-stage approach ^20^.

To evaluate our own outcomes, we examined 665 consecutive boys who underwent hypospadias repair at The Children's Hospital of Philadelphia (CHOP) from 1996 to 2006 ^1^. At a median follow up of 6.5 months, our complication rate for all repairs was 17%, defined as any post-surgical concern that warranted surgical repair; 579 of 665 (87%) boys had midshaft or distal hypospadias, while 13% had proximal hypospadias, defined by a urethral meatus proximal to the midshaft after penile degloving in the operating room. A disproportionate number of our complications (35%) occurred in the 86 (13%) boys with proximal hypospadias, with a proximal hypospadias complication rate of 39/86 (45%). This subset contrasts sharply with our 17% overall complication rate and clearly delineates one of our concerns about the hypospadias literature in that a dilution effect occurs when one groups proximal hypospadias (with poor outcomes) with distal hypospadias (more favorable outcomes). Distal repairs, with inherently good results in contemporary series, artificially inflate the outcomes for proximal repairs when these boys are grouped together. We therefore argue that proximal hypospadias warrants consideration as a separate disorder when considering surgical outcomes owing to the severity of the phenotype and the higher post-surgical complication rate.

To further examine this potential dilution effect on published results, we assessed the literature to determine the quality of proximal hypospadias publications. We conducted a PubMed search with the keyword “hypospadias”. The search identified 3492 papers published since 1995. After removing reviews, redundant studies from single institutions, and case reports, 214 were unique, peer-reviewed studies about hypospadias repair. Of the 214, 163 focused on distal hypospadias, while 51 were dedicated to proximal hypospadias. We next screened manuscripts to exclude studies with fewer than 50 patients and fewer than 2 years of follow up. While the majority of urethrocutaneous fistulas will be identified within the first year of follow up, we selected at least 2 years to examine additional complications that might not be otherwise captured in the early postoperative period ^17^. This left 32 manuscripts with a median follow up of greater than 2 years and 23 with more than 50 patients. Further refinement to include only studies with more than 50 proximal hypospadias patients and at least 2-year median follow up yielded only 11 studies. This lack of quality data makes it difficult to critically examine our surgical approach to allow for technical improvement ^2, 3, 21^.

Thankfully, this trend of underreporting is changing with three recent publications that reviewed a segregated large series of proximal hypospadias repair. Surgeons from Texas Children’s Hospital presented their 11-year experience with 56 boys with proximal hypospadias at a median follow up of 34 months ^3^. The surgeons used a two-staged repair, and their overall complication rate was 68%, defined as any additional procedures required beyond the initial planned two-stage repair. In a similar fashion, surgeons from Boston Children’s Hospital presented their results over a 20-year period for 134 boys undergoing a staged repair for proximal hypospadias. They reported a complication rate of 49% at a median follow up of 46 months, including fistula, diverticulum, meatal stenosis, and glans dehiscence ^21^. Pippi Salle *et al*. from Toronto were able to compare their experience with three separate techniques used for 140 boys with proximal hypospadias: a long TIP, dorsal inlay graft, and a staged repair ^2^. At a mean follow up ranging from 30 to 48 months, the complication rate was highest for a long single-stage TIP (53%) and lowest for the staged repair (32%). Reviewing our own experience from 2006 to 2014 with proximal hypospadias repair at CHOP corroborates these results. Of 167 consecutive patients, 86 underwent a single-stage repair and 81 a planned two-stage repair with median follow up of 29 and 31 months, respectively. The complication rate was higher for the single-stage vs. staged repair (62% vs. 49%, p=0.11), although this did not achieve significance ^1^. These numbers are much higher than historical complication rates for proximal hypospadias that were reported as low as 15–30%. Larger numbers of patients and longer follow up contribute to complication rates as high as 50–70%. Now that we have identified this discrepancy, we need to determine if this is due to the disease process itself, specifically the degree of hypoplastic penile tissue, or inadequate surgical technique.

While many boys will have their complications corrected with one additional procedure, some require multiple complex procedures to correct the sequelae of a failed initial repair and are categorized as a so-called hypospadias cripple, a designation which carries significant morbidity ^22^. Particular attention must be given to avoid this unfortunate outcome.

## Factors contributing to a high complication rate

At baseline, the hypospadiac penis is abnormal compared to unaffected boys. Patients with successful repairs typically complain of shortened penile length that correlates with increasing severity of hypospadias ^23, 24^. The corpus cavernosum and the erectile bodies of the penis are smaller, and the elasticity of the corporal tissues is compromised compared to controls ^25^. Given the hypoplastic nature of these tissues, the growth potential of the reconstructed penis and urethra is unclear and can complicate any repair. Although one recent report found an improvement in the force of the urinary stream as boys entered puberty, the full impact of penile reconstruction needs to be characterized and will be achieved only with additional long-term follow up ^26^. As these boys progress through puberty and experience exponential penile growth, previously unidentified concerns such as poor cosmetic outcome or persistent chordee may worsen ^24, 27^.

Some technical components have emerged as risk factors. Aggressive urethral mobilization for proximal TIP repair increases risk for ischemia-induced urethral stricture ^18^. Urethral diverticula occur in 4–12% of boys in whom the preputial island onlay technique is utilized for proximal hypospadias repair ^26, 28^. Persistent chordee and unsatisfactory cosmetic appearance are two less commonly reported concerns that are gaining recognition with longer follow up ^29, 30^. Delayed repair is not a good option, as results of primary hypospadias repair in adult patients are poor, approaching 50% even for distal repairs ^31, 32^.

A small glans size, particularly when the width is 14 mm or less, increases the risk of complication ^33, 34^. This is likely technical in nature owing to the placement of undue tension on the glans closure, leading to glans dehiscence, meatal stenosis, and/or urethral stricture, although the exact etiology has yet to be elucidated. Supplemental testosterone increases glans width prior to surgery, potentially reducing this risk ^35– 37^. Although a recent report disputed the significance of glans size and risk of complication ^38, 39^, smaller glans size presents a challenge in the operating room. Preoperative testosterone use should be studied in a randomized, prospective study to determine its role in surgical outcomes, as its exact benefit remains to be elucidated in a satisfactory fashion ^40^. At CHOP, it is our practice to apply intramuscular testosterone 6 and 3 weeks prior to surgery to augment glans size if the preoperative measurement is 14 mm or less.

The duration of follow up has become an increasingly important entity in hypospadias repair. Only 50% of complications are identified in the first postoperative year, and longer follow up has universally yielded higher rates of complications ^17, 41, 42^. Spinoit *et al*. examined 474 primary hypospadias repairs, of which only 54/114 (47%) of their complications were identified and operated upon within 1 year of surgery ^41^. On the other hand, 88/114 (77%) had undergone an additional procedure within the first 36 months. In a similar study, Grosos *et al*. reported that only 57% of their complications were discovered during the first year of follow up ^17^. The type of complication varied according to the time to presentation, with fistulas occurring more commonly in the first year, while urethral stenosis was more likely beyond this time point. The authors theorized that the immature ventral urethral plate displays differential growth compared to the surrounding penile tissue, which can lead to tethering as the penis grows with age. These two papers clearly indicate that follow up for less than 1 year is inadequate. We strongly agree and argue that structured follow up must extend into puberty. We simply cannot rely upon patient and parental identification of postoperative issues but instead must be invested in ensuring that the tissues have healed appropriately, are growing in proportion with the patient, and are functioning properly as these boys enter adulthood.

Options to correct penile curvature include ventral penile lengthening with corporoplasty using corporal grafts taken from native homografts (dermal, tunica vaginalis graft), extracellular matrix (SIS), ventral corporal incisions or so called “fairy cuts”, or dorsal shortening with corporal plication ^43^. In a series of 100 boys operated upon in Toronto, Braga *et al*. found an increased rate of recurrent penile curvature following dorsal plication when compared with corporal grafting (28% vs. 9%, p=0.03) ^44^. Severe chordee, defined as penile curvature greater than 30 degrees, can be debilitating from both a urinary and a sexual function standpoint when it persists or recurs after primary repair ^45^. Residual chordee occurs when the corrective procedure inadequately addressed the curvature at the initial procedure, while recurrent curvature appears because of disproportional corporal growth and may worsen as these boys progress through the exponential penile growth phase of puberty ^44, 46^. We believe that over application of the easier, dorsal plication technique in a single-stage hypospadias repair is contributing to the development of recurrent curvature as these boys age. An additional complicating factor is that it is currently unknown whether or not our current method of intraoperative assessment of chordee in the pre-pubertal penis correlates well with the ultimate post-pubertal appearance of the penis. All of these factors have led to us now favoring corporoplasty to lengthen the ventral penile shaft to fully correct penile curvature, even though this requires two procedures. Long-term results quantifying rates of residual curvature, aneurismal dilation of the corporal graft, and the possibility for erectile dysfunction still need to be addressed, although to date we have not seen these concerns.

At CHOP, we find that boys with persistent penile curvature after primary repair, in both pre- and post-pubertal age groups, are a particularly challenging group owing to their increased age and scarring following previous surgery. Our approach to recurrent chordee has evolved and now includes a series of procedures designed to first straighten the penis, usually with ventral lengthening via corporal grafting with supplemental dartos and skin coverage. Buccal mucosa is then placed into this soft tissue bed as a substrate for urethral reconstruction 1 year later. The urethra is reconstructed 1 year later, and to provide adequate skin coverage we utilize a modified Cecil procedure. Finally, separation of the Cecil flap after 1 additional year results in supple penile tissue, allowing us to consistently achieve an acceptable outcome ^22, 47, 48^. This 4-year process requires a significant amount of investment from the patient’s perspective but highlights our concern and the need to avoid such outcomes.

Why are proximal repairs harder? In addition to the presence of immature penile tissue with potentially compromised healing potential, the longer urethroplasty required to repair a proximal hypospadias poses inherent risk. The surgically constructed urethra does not expand during voiding as would a normal urethra; therefore, an anatomically appropriate diameter tube reconstructed from buccal or skin tissue will not convey urine as a normal urethra would. A long neourethra more dramatically demonstrates the physics behind laminar flow and fluid dynamics. According to Poiseuille’s law, the resistance to flow in a cylinder is proportional to the length of the tube but is inversely proportional to the radius to the fourth power. In plain terms, the pressure required to push urine through the lumen of the urethra directly increases with the length of the tube. At the same time, minor variations to the radius, either increasing or decreasing in size, will have a much greater impact upon intraluminal pressure. The longer the tube, the greater the risk for stricture development and/or a failure of the reconstructed urethra to expand with voiding, increasing resistance to urine flow, ultimately resulting in fistula and/or urethral diverticulum formation ^49^.

## The future

Can we get better at proximal hypospadias repair? First we need to develop a standardized system designed to quantify the severity of the hypospadias. Doing so would create a universal hypospadias language that would facilitate collaboration across institutions to aid in patient recruitment, the development of new techniques, and rigorous outcome evaluation. Grading systems based on the location of the urethral meatus have been inconsistent and have prevented clear comparison of series from different centers. The GMS (glans meatus shaft) score adds precision to hypospadias scoring but is still gaining popularity and will require future validation ^50^. It incorporates factors such as glans width, degree of penile curvature, and quality of urethral plate to generate a severity score for each boy preoperatively and postoperatively ^34^. We are participating in a nationwide effort led by the Society for Pediatric Urology workgroup whose focus is to standardize the perioperative assessment of patients with hypospadias to objectify the patient phenotype to add precision to staging, which will lead to the potential for true nationwide comparisons.

In the past, we have not always measured the patient’s and family’s impressions of the repair. Parental and patient perception of outcomes after surgery does not always match the surgeon’s impression of their work ^51, 52^. The penile perception score has demonstrated an ability to bridge this deficit ^53^. Additional scoring systems include the HOPE and HOSE scoring systems ^54– 56^. As surgeons, we need to determine if our evaluation of a sufficient location of the urethral meatus, the cosmetic appearance of the glans, and the degree of redundant skin correlates with patient perception or if other factors are more important for patient satisfaction, which, in the end, is the key component of a successful repair ^30^. These tools should facilitate this and will be a standard component of patient follow up.

What methods can we use to improve? Our participation in the Multi-Institution Bladder Exstrophy Consortium (MIBEC) has advanced our understanding of the surgery for bladder exstrophy ^57^. In this system, surgeons come together to coach, standardize, and carefully record complex surgery surrounding bladder exstrophy repair. Coaches are common in athletics. Editors are critical to the writing process. Conductors help organize and improve musical performance. We believe this approach can be applied to surgery, particularly for the rare or complex challenges such as proximal hypospadias, to improve our approach and outcomes ^57, 58^. Opportunities for coaching are plentiful and may include informal collaboration amongst onsite partners and colleagues, but in our experience organized participation from outside teams can be particularly effective ^57^. Current technology such as live streaming and high-definition video cameras facilitates collaboration across institutions. The environment of discussion and open sharing of results and techniques, particularly for a relatively rare disease process such as proximal hypospadias, will increase exposure and advance our understanding.

We now assess and assign a standard risk score in the preoperative, intraoperative, and postoperative period. Our data points include objective measurements of glans and urethral plate width, urethral meatus location, the degree of chordee, the length of the neourethra, suture utilization, and surgical techniques. Measurements are carefully made with a caliper. Chordee is precisely measured using a goniometer. Follow up will extend beyond puberty. A family satisfaction score that incorporates patient and family satisfaction will supplement our impressions and ensure that we are indeed doing good work when we think we are. Then we will be able to make recommendations for these complex patients, such as proceeding with a staged vs. single-stage repair, delayed glansplasty, a prolonged urethral stent, supplemental testosterone, etc., in hopes of further reducing complications.

## Conclusions

Proximal hypospadias is a challenging surgical entity, the degree of which has only recently been exposed in the literature. By appropriately staging each boy, we will facilitate collaboration in order to optimize the surgical approach and to assess outcomes across multiple institutions. This practice will allow us to identify risk factors for failure and pursue approaches that will improve success. We realize that an algorithm for hypospadias management is unrealistic given its complex nature. Nevertheless, the accumulated data will help guide us toward more successful approaches, such as deciding to proceed with a staged repair, the appropriate method to correct chordee, and the utilization of testosterone to increase glans size. With these efforts, we can hope to improve upon the current success rates that we are achieving for these boys.

## Abbreviations

CHOP, The Children’s Hospital of Philadelphia; TIP, tubularized incised plate urethroplasty.
